
JOURNAL INFORMATION
==============================
NLM Title Abbreviation: Access Microbiol
Journal ID: Access Microbiol
NLM Journal ID: 101746927
Journal ID: acmi

Access Microbiology

EISSN: 2516-8290
Publisher: Microbiology Society

ARTICLE INFORMATION
==============================
PMCID: PMC7491928
PMID: 32974496
Article version: 1
Subjects: Poster

Abstracts from the International Meeting on Arboviruses and their Vectors 2019

Publication date: 2019
Electronic publication date: 2019 Dec 20
Volume: 1
Issue: 10
Electronic Location ID: 4
Copyright: © 2019 The Authors
Copyright year: 2019
License: This is an open-access article distributed under the terms of the Creative Commons Attribution License.
License URL: https://creativecommons.org/licenses/by/4.0/

==============================


Mapping the virome in lab-reared and wild-caught Aedes albopictus mosquitoes

http://jmmcr.microbiologyresearch.org

Zhao Lu
Atoni Evans
Shi Chenyan
Yuan Zhiming
Xia Han *
Wuhan Institute of Virology , Chinese Academy of Sciences , Wuhan
University of Chinese Academy of Sciences , Beijing , China
Department of Microbiology and Immunology , Laboratory of Viral Metagenomics , Rega Institute for Medical Research Leuven
*Correspondence: Han Xia, hanxia@wh.iov.cn

A salivary protein of Aedes aegypti promotes dengue-2 virus replication and transmission

http://jmmcr.microbiologyresearch.org

Shiao Shin-Hong *
National Taiwan University , Taipei , Taiwan
*Correspondence: Shin-Hong SHIAO, shshiao@ntu.edu.tw

A novel class of small molecule inhibitors targeting the chikungunya virus capping machinery with a high barrier to resistance

http://jmmcr.microbiologyresearch.org

Abdelnabi Rana *
Kovacikova Kristina
Kirchebner Julia
Leyssen Pieter
Marchand Arnaud
Chaltin Patrick
Puerstinger Gerhard
Langer Thierry
Quérat Gilles
Coutard Bruno
Hemert Martijn van
Neyts Johan
Delang Leen
KU Leuven , Rega Institute for Medical Research , Leuven
Leiden University Medical Center , Leiden , Netherlands
University of Innsbruck , Innsbruck , Austria
Centre for Drug Design and Discovery , KU Leuven , Leuven
University of Vienna , Vienna , Austria
Aix Marseille Université , Marseille , France
*Correspondence: Rana Abdelnabi, rana.abdelnabi@kuleuven.be

The piRNA pathway in host-pathogen interaction: Aedes albopictus and arboviruses

http://jmmcr.microbiologyresearch.org

Marconcini Michele *
Houé Vincent
Valerio Federica
Palatini Umberto
Pischedda Elisa
Ometto Lino
Forneris Federico
Failloux Anna-Bella
Bonizzoni Mariangela
Chávez Alejandro Nabor Lozada
Department of Biology and Biotechnology , University of Pavia , Pavia
Institut Pasteur , Paris , France
*Correspondence: Michele Marconcini, michele.marconcini01@ateneopv.it

Discovery of novel endogenous viral elements in Aedes spp. mosquitoes

http://jmmcr.microbiologyresearch.org

Pischedda Elisa *
Palatini Umberto
Crava Maria Cristina
Mattia Annamaria
Bonizzoni Mariangela
University of Pavia , Pavia , Italy
*Correspondence: Elisa Pischedda, elisa.pischedda01@universitadipavia.it

Assessment of tick-borne flavivirus host factors through genome-scale screens

http://jmmcr.microbiologyresearch.org

Flather Dylan P. *
Marceau Caleb D.
Mlera Luwanika
Grabowski Jeffrey M.
Bloom Marshall E.
Rocky Mountain Laboratories , NIAID , NIH
NGM Biopharmaceuticals , South San Francisco , USA
The BIO5 Institute , University of Arizona , Tucson
*Correspondence: Dylan Flather, dylan.flather@nih.gov

Molecular determinants of dengue virus infection in Aedes aegypti midgut

http://jmmcr.microbiologyresearch.org

Siriphanitchakorn Tanamas *
Choy Milly M.
Ng Wy Ching
Ng Dorothy
Tan Hwee Cheng
Zhang Lixin Summer
Kini R. Manjunatha
Ooi Eng Eong
Programme in Emerging Infectious Diseases , Duke-NUS Medical School , Singapore
Department of Biological Sciences , National University of Singapore , Singapore
Department of Microbiology and Immunology , Yong Loo Lin School of Medicine , National University of Singapore
Saw Swee Hock School of Public Health , National University of Singapore , Singapore
*Correspondence: Tanamas Siriphanitchakorn, t.siriphanitchakorn@u.nus.edu

Establishment of a stable subgenomic dengue virus type 1 replicon system in Aedes albopictus mosquito cells for identification of DENV transmission blocking molecules

http://jmmcr.microbiologyresearch.org

Patel Sameera *
Besson Benoit
Rij Ronald van
Dechering Koen
TropIQ Health Sciences B.V. , Nijmegen , Netherlands
Dept. Medical Microbiology , Radboud Institute for Molecular Life Sciences , Nijmegen
*Correspondence: Sameera Saheb Pasha Patel, sameera.patel@radboudumc.nl

National Collection of Pathogenic Viruses: A Repository for Well Characterised and Authenticated Viruses

http://jmmcr.microbiologyresearch.org

Onianwa Okechukwu *
Russell Julie
Alexander Sarah
Public Health England , Salisbury , United Kingdom
*Correspondence: Okechukwu Onianwa, okechukwu.onianwa@phe.gov.uk

DENV-captured plasmin enhances mosquito midgut infection and is inhibited by an endogenous Kazal-type inhibitor AaTI

http://jmmcr.microbiologyresearch.org

Mok Yu Keung *
Ramesh Karthik
Wong Benjamin
Sayed Ahmed
Misse Dorothee
Manjanatha Kini
Pompoon Julien
National University of Singapore , Singapore , Singapore
Duke-NUS Medical School , Singapore , Singapore
Université de Montpellier , Montpellier , France
*Correspondence: Yu Keung Mok, dbsmokh@nus.edu.sg

Glucose regulated protein 78 (GRP78) interacts with Zika virus envelope and is required for a productive infection

http://jmmcr.microbiologyresearch.org

Royle Jamie *
Ramirez-Santana Carolina
Donald Claire
Akpunarlieva Snezhana
Gestuveo Rommel
Anaya Juan-Manuel
Burchmore Richard
Kohl Alain
Varjak Margus
MRC -University of Glasgow , Centre for Virus Research , Glasgow
Center for Autoimmune Diseases Research , Bogota , Colombia
Glasgow Polyomics Facility , Glasgow , United Kingdom
*Correspondence: Jamie Royle, j.royle.2@research.gla.ac.uk

Pan-antivirals to combat re-emerging alphaviruses

http://jmmcr.microbiologyresearch.org

Jacobs Sofie *
Abdelnabi Rana
Lambin Dominique
Marchand Arnaud
Chaltin Patrick
Neyts Johan
Delang Leen
KU Leuven , Leuven , Belgium
Rega Institute for Medical Research , Leuven , Belgium
Cistim , Leuven , Belgium
Centre for Drug Design and Discovery , Leuven , Belgium
*Correspondence: Sofie Jacobs, sofie-jacobs@kuleuven.be

Biotyping of TBEV-infected IRE/CTVM19 tick cell line

http://jmmcr.microbiologyresearch.org

Loginov Dmitry *
Böttinger Katharina
Loginova Yana
Dycka Filip
Sterba Jan
University of South Bohemia , České Budějovice , Czech Republic
Institute of Parasitology , Biology Centre of the Czech Academy of Sciences , České Budějovice
Orekhovich Institute of Biomedical Chemistry , Moscow , Russian Federation
*Correspondence: Dmitry Loginov, dloginov@jcu.cz

Chikungunya virus resistant to the antiviral favipiravir is severely attenuated in mosquitoes

http://jmmcr.microbiologyresearch.org

Langendries Lana
Jacobs Sofie
Abdelnabi Rana
Neyts Johan
Delang Leen *
Rega Institute for Medical Research , KU Leuven , Leuven
*Correspondence: Leen Delang, leen.delang@kuleuven.be

Lumpy skin disease virus does not replicate productively in insect cell lines

http://jmmcr.microbiologyresearch.org

Cook Charlotte *
Dietrich Isabelle
Greaves David
Darpel Karin
Beard Pip
The Pirbright Institute , Woking , United Kingdom
The University of Oxford , Oxford , United Kingdom
*Correspondence: Charlotte Cook, charlotte.cook@pirbright.ac.uk

Posaconazole is a novel inhibitor for alphavirus viral entry

http://jmmcr.microbiologyresearch.org

Varghese Finny S
Meutiawati Febrina *
Teppor Mona
Merits Andres
Rij Ronald P van
Radboud Institute for Molecular Life Sciences , Radboudumc , Nijmegen
Institute of Technology , University of Tartu , Tartu
*Correspondence: Febrina Meutiawati, febrinameutia@gmail.com

Karyotype changes in cultivated tick cell lines

http://jmmcr.microbiologyresearch.org

Kotsarenko Kateryna *
Vechtova Pavlina
Sterba Jan
University of South Bohemia , Ceske Budejovice , Czech Republic
Biology Centre CAS , Ceske Budejovice , Czech Republic
*Correspondence: Kateryna Kotsarenko, kkotsarenko@prf.jcu.cz

Targeting functional RNA structures in CHIKV and ZIKV

http://jmmcr.microbiologyresearch.org

Prosser Oliver *
Merits Andres
Stonehouse Nicola J
Tuplin Andrew
University of Leeds , Leeds , United Kingdom
University of Tartu , Tartu , Estonia
*Correspondence: Oliver Prosser, bs12odp@leeds.ac.uk

Interrogation of the CHIKV nsP-host interactome in human and mosquito cells

http://jmmcr.microbiologyresearch.org

Bartholomeeusen Koen *
Groof Susan De
Hoffmann Chris
Agombin Terence
Caluwé Lien De
Coppens Sandra
Daled Simon
Deforce Dieter
Ariën Kevin
Insitute of Tropical Medicine , Antwerp , Belgium
UGent , Gent , Belgium
*Correspondence: Koen Bartholomeeusen, kbartholomeeusen@itg.be

Development of reverse genetics for Toscana virus

http://jmmcr.microbiologyresearch.org

Alexander Akira *
Dunlop James
Brennan Ben
Kuchi Srikeerthana
Mair Daniel
Filipe Ana da Silva
Ratinier Maxime
Arnaud Frédérick
Kohl Alain
MRC Centre for Virus Research , Glasgow , United Kingdom
Infections virales et pathologie comparée (IVPC) , Lyon , France
*Correspondence: Akira Alexander, akira.alexander@glasgow.ac.uk

The Basigin (CD147)-CD98 protein complex is involved in Chikungunya virus attachment and entry in human cells

http://jmmcr.microbiologyresearch.org

Caluwé Lien De *
Coppens Sandra
Daled Simon
Dhaenens Maarten
Deforce Dieter
Ostade Xaveer Van
Ariën Kevin
Bartholomeeusen Koen
Institute of Tropical Medicine , Antwerp , Belgium
Progentomics , Gent , Belgium
University of Antwerp , Antwerp , Belgium
*Correspondence: Lien De Caluwé, ldecaluwe@itg.be

Mosquito vector factors driving arbovirus infection in Anopheles

http://jmmcr.microbiologyresearch.org

Loreto Raquel *
Failloux Anna-Bella
Vernick Kenneth
Institut Pasteur , Paris , France
*Correspondence: Raquel Loreto, raquel.gontijo-de-loreto@pasteur.fr

Louping Ill Virus Outbreak in North-East Wales

http://jmmcr.microbiologyresearch.org

Dorey-Robinson Daniel
Folly Arran
Phipps Paul
Vidana Beatriz
Holmes Paul
Johnson Nicholas *
Animal and Plant Health Agency , Addlestone , United Kingdom
Animal and Plant Health Agency , Shrewsbury , United Kingdom
University of Surrey , Guildford , United Kingdom
*Correspondence: Nicholas Johnson, nick.johnson@apha.gov.uk

Identification of candidate molecular determinants of the vector competence of Ixodes ricinus for members of the tick-borne encephalitis complex

http://jmmcr.microbiologyresearch.org

Lemasson Manon *
Caignard Grégory
Unterfinger Yves
Attoui Houssam
Bell-Sakyi Lesley
Moutailler Sara
Bonnet Sarah
Vitour Damien
Richardson Jennifer
Lacour Sandrine A.
UMR 1161 Virologie (ANSES-INRA-ENVA) , Maisons-Alfort , France
Department of Infection Biology , Institute of Infection and Global Health , University of Liverpool
UMR 956 Bipar (ANSES-INRA-ENVA) , Maisons-Alfort , France
*Correspondence: Manon Lemasson, manon.lemasson@vet-alfort.fr

Monitoring of Insecticide Resistance on Aedes sp. Mosquitoes in Banyumas Regency, Indonesia

http://jmmcr.microbiologyresearch.org

Wijayanti Siwi Pramatama Mars *
Octaviana Devi
Nurlaela Sri
Widiastuti Dyah
Department of Public Health , Jenderal Soedirman University , Purwokerto
Research and Development Unit for Zoonosis Control , Banjarnegara , Indonesia
*Correspondence: Siwi Pramatama Mars Wijayanti, mars_manuel@yahoo.com

Salivary gland RNA-seq from arbovirus-infected Aedes aegypti and Aedes albopictus provides insights into virus transmission

http://jmmcr.microbiologyresearch.org

Modahl Cassandra *
Chowdhury Avisha
Oliveira Fabiano
Kini R. Manjunatha
Pompon Julien
National University of Singapore , Singapore , Singapore
National Institute of Allergy and Infectious Diseases , Bethesda , Maryland
Duke-NUS Medical School , Singapore , Singapore
4MIVEGEC , IRD , CNRS
*Correspondence: Cassandra Modahl, dbscmm@nus.edu.sg

Transcriptomic analysis of human neurons and astrocytes infected with TBEV strains of different virulence

http://jmmcr.microbiologyresearch.org

Selinger Martin *
Vechtova Pavlina
Tykalova Hana
Sterba Jan
Grubhoffer Libor
Institute of Parasitology , Biology Centre CAS , Ceske Budejovice
Faculty of Science , University of South Bohemia , Ceske Budejovice
*Correspondence: Martin Selinger, selinger@paru.cas.cz

Transcriptional and translation shut-off in TBEV infected neural cells and involvement of viral C protein

http://jmmcr.microbiologyresearch.org

Tykalová Hana *
Selinger Martin
Štěrba Ján
Věchtová Pavlína
Kohl Alain
Schnettler Esther
Grubhoffer Libor
Faculty of Science , University of South Bohemia in Ceske Budejovice , Branisovska 1760
Institute of Parasitology , Biology Centre CAS , Branisovska 31
MRC-University of Glasgow Centre for Virus Research , Glasgow G61 1QH , Glasgow
Bernhard Nocht Institute for Tropical Medicine , Bernhard-Nocht-Str. 74 , 20359 Hamburg
German Centre for Infection Research (DZIF) , partner site Hamburg-Luebeck-Borstel-Riems , 20359 Hamburg
*Correspondence: Hana Tykalová, tykalova@paru.cas.cz

The Using of Serum Free Media for the Production of J93-463-1-16-10 Monoclonal Antibody against Japanese encephalitis virus

http://jmmcr.microbiologyresearch.org

Tangkananond Wipa *
Petchin Nattha
Department of Biotechnology , Faculty of Science and Technology , Thammasat University
*Correspondence: Wipa Tangkananond, wipatang@tu.ac.th

The Cryopreservation of Cos Lettuce Callus by Using Liquid Nitrogen

http://jmmcr.microbiologyresearch.org

Tangkananond Wipa *
Department of Biotechnology , Faculty of Science and Technology , Thammasat University
*Correspondence: Wipa Tangkananond, wipatang@tu.ac.th

Toolkit for the study of yellow fever virus

http://jmmcr.microbiologyresearch.org

Velazquez Ricardo Sanchez *
Lorenzo Giuditta De
Tandavanitj Rapeepat
Setthapramote Chayanee
Varjak Margus
Kohl Alain
Patel Arvind
MRC Centre for Virus Research , University of Glasgow , Glasgow
*Correspondence: Ricardo Sanchez Velazquez, r.sanchez-velazquez.1@research.gla.ac.uk

The Tick Cell Biobank: new arthropod cell lines for arbovirus research

http://jmmcr.microbiologyresearch.org

Bell-Sakyi Lesley *
Hartley Catherine
Darby Alistair
Baylis Matthew
Makepeace Benjamin
Institute of Infection and Global Health , University of Liverpool , Liverpool
Institute of Integrative Biology , University of Liverpool , Liverpool
*Correspondence: Lesley Bell-Sakyi, l.bell-sakyi@liverpool.ac.uk

AurKB activity is necessary for Dengue virus release

http://jmmcr.microbiologyresearch.org

Olais Jose Humberto Perez
Jimenez Fernando Ruiz *
García Esther Joquebeb Calderón
Rivas Rosaura Hernández
Angel Rosa Maria Del
CINVESTAV , Mexico city , Mexico
Nottingham University , Nottingham , Nottingham
*Correspondence: Fernando Ruiz Jimenez, mbxfr2@nottingham.ac.uk

Implications of vertical transmission of Alphaviruses in Aedes aegypti mosquitoes

http://jmmcr.microbiologyresearch.org

Rodriguez Julio *
Fazakerley John
The Peter Doherty Institue - University of Melbourne , Melbourne , Australia
Faculty of Agricultural and Veterinary Sciences- University of Melbourne , Melbourne , Australia
*Correspondence: Julio Rodriguez, julio.rodriguez@unimelb.edu.au

JNK pathway-a key mediator of antiviral immunity in mosquito salivary glands

http://jmmcr.microbiologyresearch.org

Chowdhury Avisha *
Modahl Cassandra M.
Tan SiokThing
Wong Benjamin
Kini R. Manjunatha
Pompon Julien
Biological sciences , National University of Singapore , Singapore
Emerging Infectious Diseases , Duke-NUS Graduate Medical School , Singapore
MIVEGEC , IRD , CNRS
*Correspondence: Avisha Chowdhury, dbsavis@nus.edu.sg

The effect of co-infecting Anaplasma phagocytophilum on replication of Langat Virus, a model for Louping ill virus, in Ixodes spp. tick cells

http://jmmcr.microbiologyresearch.org

Luu Lisa *
Bradley George
Al-Khafaji Alaa
Bell-Sakyi Lesley
University of Liverpool , Liverpool , United Kingdom
*Correspondence: Lisa Luu, lisaluu@liverpool.ac.uk

The emerged genotype I of Japanese encephalitis virus shows an infectivity similar to genotype III in Culex pipiens mosquitoes

http://jmmcr.microbiologyresearch.org

Hameed Muddassar *
Shanghai Veterinary Research Institute , Shanghai , China
*Correspondence: Muddassar Hameed, mudasar386@gmail.com

A Viral Metagenomic Analysis Reveals Rich Viral Abundance and Diversity in Mosquitoes from Pig Farms

http://jmmcr.microbiologyresearch.org

Wahaab Abdul *
Shanghai Vetrinary Research Institute , Chinese Academy of Agriculture Sciences , Shanghai
*Correspondence: ABDUL WAHAAB, abdul.wahaab@uaf.edu.pk

The Expression of Japanese Encephalitis Virus Envelope Gene in Green Cos

http://jmmcr.microbiologyresearch.org

Tangkananond Wipa *
Lomonossoff George
Department of Biotechnology , Faculty of Science and Technology , Thammasat University
Department of Biological Chemistry , John Innes Centre , Colney lane
*Correspondence: Wipa Tangkananond, wipatang@tu.ac.th

Zika virus infection of glia leads to secondary injury to axons and dendrites

http://jmmcr.microbiologyresearch.org

Schultz Verena *
Crawford Colin
Barrie Jennifer
Cumberworth Stephanie
Donald Claire
Barnett Susan
Linington Christopher
Kohl Alain
Willison Hugh
Edgar Julia
University of Glasgow , Glasgow , United Kingdom
*Correspondence: Verena Schultz, verena.schultz@glasgow.ac.uk

Of Mice and Monkeys: Determining Protective Serological Titres in Model Zika Virus Infections

http://jmmcr.microbiologyresearch.org

Ferguson Debbie *
Kempster Sarah
Dowall Stuart
Ham Claire
Hall Jo
Mattiuzzo Giada
Graham Victoria
Findlay-Wilson Stephen
Berry Neil
Page Mark
Hewson Roger
Almond Neil
IDD-NIBSC , South Mimms , United Kingdom
PHE , Porton Down , United Kingdom
Virology-NIBSC , South Mimms , United Kingdom
*Correspondence: Debbie Ferguson, deborah.ferguson@nibsc.org

Stage specific transcriptome profiling of castor bean tick Ixodes ricinus

http://jmmcr.microbiologyresearch.org

Vechtova Pavlina *
Füssy Zoltan
Sterba Jan
Cegan Radim
Benes Vladimir
Grubhoffer Libor
Faculty of Science , University of South Bohemia , Ceske Budejovice
Institute of Parasitology , Biology Centre CAS , Ceske Budejovice
Institute of Biophysics of the CAS , v.v.i. , Brno
Genomics Core Facility , EMBL , Heidelberg
*Correspondence: Pavlina Vechtova, vechtova@prf.jcu.cz

Lumpy skin disease virus: transmission to dipteran vectors using animal and ex-vivo models

http://jmmcr.microbiologyresearch.org

Bernardo Beatriz Sanz *
Suckoo Rey
Darpel Karin
Hawes Pippa
Basu Sanjay
Langlands Zoe
Gubbins Simon
Beard Pip
The Pirbright Institute , Woking , United Kingdom
*Correspondence: Beatriz Sanz Bernardo, beatriz.sanz-bernardo@pirbright.ac.uk

Zika virus utilises the ubiquitin-proteasome pathway during infection of mosquito and human cells

http://jmmcr.microbiologyresearch.org

Gestuveo Rommel
Varjak Margus
Royle Jamie
Donald Claire
Kohl Alain *
MRC-University of Glasgow Centre for Virus Research , Glasgow , United Kingdom
University of the Philippines , Iloilo , Philippines
*Correspondence: Alain Kohl, Alain.Kohl@glasgow.ac.uk

St Abbs Head phlebovirus – a separate virus species or a strain of Uukuniemi phlebovirus?

http://jmmcr.microbiologyresearch.org

Szemiel Agnieszka *
Willett Brian
Ma Wing See
MRC - University of Glasgow Centre for Virus Research , Glasgow , United Kingdom
*Correspondence: Agnieszka Szemiel, agnieszka.szemiel@glasgow.ac.uk

Alphavirus E1 fusion protein: alternative conformations of the post-fusion trimer depending on the alphavirus

http://jmmcr.microbiologyresearch.org

GRAU Nina *
MONCOQ Karine
ROUVINSKI Alexander
DUQUERROY Stéphane
HAOUZ Ahmed
VANEY Marie-Christine
REY Félix
Institut Pasteur , Paris , France
Institut de Biologie Physico-Chimique , Paris , France
Hebrew University of Jerusalem , Jerusalem , Israel
*Correspondence: Nina GRAU, nina.grau@pasteur.fr

Antagonistic action of blood-feeding and mating on the gut immunity in the female mosquito Aedes aegypti

http://jmmcr.microbiologyresearch.org

Almire Floriane *
Terry Sandra
Kean Joy
Donald Claire
Varjak Margus
McFarlane Melanie
Kohl Alain
Pondeville Emilie
MRC-University of Glasgow - Centre for Virus Research , Glasgow , United Kingdom
*Correspondence: Floriane Almire, f.almire.1@research.gla.ac.uk

